# Haplotyping the human leukocyte antigen system from single chromosomes

**DOI:** 10.1038/srep30381

**Published:** 2016-07-27

**Authors:** Nicholas M. Murphy, Matthew Burton, David R. Powell, Fernando J. Rossello, Don Cooper, Abha Chopra, Ming Je Hsieh, David C. Sayer, Lavinia Gordon, Mark D Pertile, Brian D. Tait, Helen R. Irving, Colin W. Pouton

**Affiliations:** 1 Monash Institute of Pharmaceutical Sciences, Monash University, Parkville, VIC, Australia; 2 Preimplantation Genetic Diagnosis, Melbourne IVF, Melbourne, VIC, Australia; 3 Flow Cytometry & Imaging, Murdoch Children’s Research Institute, Parkville, VIC, Australia; 4 Bioinformatics Platform, Monash University, Clayton, VIC, Australia; 5 Department of Anatomy and Developmental Biology, Monash University, Clayton, VIC, Australia; 6 Australian Regenerative Medicine Institute, Monash University, Clayton, VIC, Australia; 7 Institute for Immunology and Infectious Diseases, Murdoch University, Murdoch, WA, Australia; 8 Conexio Genomics, Fremantle, WA, Australia; 9 Australian Genome Research Facility, The Walter and Eliza Hall Institute of Medical Research, Royal Parade, Parkville, VIC, Australia; 10 Victorian Clinical Genetics Services, Parkville, VIC, Australia; 11 Department of Paediatrics, University of Melbourne, Parkville, VIC, Australia; 12 Transplant Services, Australian Red Cross Blood Service, Parkville, VIC, Australia

## Abstract

We describe a method for determining the parental HLA haplotypes of a single individual without recourse to conventional segregation genetics. Blood samples were cultured to identify and sort chromosome 6 by bivariate flow cytometry. Single chromosome 6 amplification products were confirmed with a single nucleotide polymorphism (SNP) array and verified by deep sequencing to enable assignment of both alleles at the HLA loci, defining the two haplotypes. This study exemplifies a rapid and efficient method of haplotyping that can be applied to any chromosome pair, or indeed all chromosome pairs, using a single sorting operation. The method represents a cost-effective approach to complete phasing of SNPs, which will facilitate a deeper understanding of the links between SNPs, gene regulation and protein function.

Genetic haplotyping and genome phasing are increasingly prominent components of both sequencing technologies and clinical genotyping/phenotyping. Chromosomal phasing or haplotype phasing refers to the physical linkage of chromosomal polymorphisms unencumbered by the ambiguity of polyploidy. The effect of phase and the quantifiable effects that the phasing of inter-chromosomal single nucleotide polymorphisms (SNPs), copy number variations (CNVs), insertions/deletions (INDELs) and structural rearrangements have on the expression of adjacent co-inherited genes is a challenging but increasingly crucial component of contemporary medicine. We sought to adapt published methods using flow cytometry to sort chromosomes in suspension. This allowed us to acquire single chromosomes and to rapidly determine the haplotype phase of genes which are of critical importance to immune function in bone-marrow transplantation^1^.

Located on the short arm of human chromosome 6, the major histocompatibility complex (MHC) has been extensively haplotype sequenced due to its relevance to transplant matching and disease diagnosis^2^. The highly polymorphic human leukocyte antigen (HLA) system genes are central to the recognition of self from non-self. For this reason, the matching of HLA loci sequences is a cornerstone of donor/recipient matching in organ and haematopoietic stem cell transplantation (HSCT)^3^.

During antigen presentation, the MHC Class I genes (*HLA-A*, *HLA-B* and *HLA-C*) present cytosolic peptides and MHC Class II genes (*HLA-DRB1*, *HLA-DQA1*, *HLA-DQB1*, *HLA-DPA1* and *HLA-DPB1*) present extracellular peptides. Matching alleles of MHC Class I and II is standard, and there is also growing interest in selecting killer immunoglobulin-like receptor (KIR) alleles^4^,^5^,^6^. While donor/recipient mismatches are tolerated in solid organ transplants, matching in HSCT is stringent, due to increasing severity of graft versus host disease with rising numbers of HLA mismatches. HLA genes are co-dominant and are inherited as conserved ancestral haplotypes from each parent^7^. At present the definitive method of generating haplotype data on an individual is by family pedigree^8^. The demand for a high degree of matching has led to the establishment of HSCT donor registries worldwide which contain HLA data on some 16 million potential donors. Despite the technical challenge of HLA haplotyping, there is evidence that HLA haplotype matching decreases acute Graft-versus-host-disease (GvHD) without modifying risk of relapse, possibly due to non-HLA alleles on the haplotype, and can increase survival in unrelated transplants^9^,^10^,^11^. It is not feasible to establish which donors are haplotype-matched with a particular patient, therefore current recruitment and HLA typing of donors on to HSCT registries does not include definition of HLA haplotypes. Resolving the sequence of each HLA allele directly from diploid genomic DNA requires a significant sequencing campaign and presents a complex computational problem. To date, the most successful HLA-typing approaches employ targeted fragment enrichment for allele calling but do not result in haplotype phasing^12^. There is a need to develop straightforward processes whereby the phase of alleles linked in series can be established to improve clinical outcomes. The ability to resolve phase may have relevance beyond HLA typing; HLA typing may also be used in healthcare areas besides transplantation, specifically in drug hypersensitivity^13^ but also more widely in disease association genomics^14^. The value of higher resolution typing will be able to be assessed with greater precision as further typing is done.

Physical separating parentally inherited homologous chromosomes simplifies sequencing in the laboratory, facilitates analysis and clinical decision-making^14^. Amplification products can undergo standard high throughput sequencing of each of the two chromosomes inherited by a single individual, or alternatively, can be genotyped directly using SNP arrays. The method can be used to haplotype any individual, avoiding the need for segregation studies through extensive family genotyping. The method described below can be applied to any chromosome pair, and we have been successful in sorting a single parental copy of all chromosomes in a single FACS experiment (data not shown). Due to the critical need of haplotyping in HSCT, we chose to exemplify the method for this manuscript using chromosome 6 (Fig. 1).

## Results

We haplotyped the most relevant HLA alleles by single chromosome isolation using bivariate flow cytometry followed by whole chromosome amplification. A prospective bone-marrow donor was HLA typed at the Victorian Transplantation and Immunogenetics Service in 2008 using genomic DNA sequence based typing^15^,^16^. The donor is heterozygous at all loci with the exception of HLA-A, thus haplotype phasing from the blood of this donor provided a convincing validation of the method. The following alleles at the HLA loci were assigned: HLA-A^*^01:01; HLA-B*08:01,*27EKN* (*27:05/*27:13); HLA-C*01:02,*07CVAG* (*07:01/*07:06/*07:18/*07:52); HLA-DRB1*01:01,*03:01; HLA-DRB3*01:01; HLA-DQB1*02:01,*05:01; HLA-DPB1*01:01,*04BDKS* (*04:02/*06:02). The chromosome suspension was prepared directly from blood. Blood cells were stimulated with phytohemagglutinin, arrested in metaphase, lysed, and the chromosomes were stabilised in a polyamine buffer. The addition of DNA base-specific intercalating dyes enabled bivariate identification of chromosomal homolog clusters with a flow cytometer. The resolution of the bivariate flow karyotype was tested one day after cell lysis and a high quality flow karyotype was obtained with each chromosome cluster clearly resolved except for the chromosome 9–12 cluster (Fig. 2a), typical for human bivariate flow karyotypes^17^. Chromosomes in suspension were stored for up to 90 days at 4 °C prior to single chromosome acquisition. The older preparations had decreased cluster resolution with overlap in the periphery of neighbouring chromosomal clusters compared to one day old preparations, but nevertheless were resolvable. Single signals of the bivariate flow karyotype chromosome 6 cluster were sorted into separate PCR tubes. In order to confirm resolution of chromosomes, 48 copies of single chromosome 6 were acquired into separate PCR tubes. Thirty six samples were amplified with the PicoPLEX whole genome amplification Kit (Rubicon Genomics, USA) and 24 samples were randomly selected for amplification. Of these, five samples were found to contain only fragments of chromosome 6. Samples were then run on 24 Sure (+) BAC microarray (BlueGnome, Cambridge) to select samples containing DNA from flow-sorted then amplified chromosome 6 samples. Five of the 24 samples contained amplified DNA from chromosome 6 without any non-chromosome 6 DNA (Fig. 2b). To determine their individual identity from one or the other parent, these samples containing chromosome 6 DNA were used for microsatellite testing to compare individual chromosome 6 samples against diploid genomic DNA. Microsatellite maker testing at the polycystic kidney and hepatic disease 1 (PKHD1) locus (6p12.2) was undertaken using a custom multiplex mix for the gene and flanking microsatellites. The donor genomic DNA contained three loci with heterozygote peaks that were suitable for determining flow-sorted-then-amplified samples containing single chromosome 6 (i.e. from one parent). Four of the flow-sorted-then-amplified chromosome 6 samples had homozygous peaks of the same size that matched one of the two dual peaks identified from diploid genomic DNA. An additional sample had homozygote peaks at sizes matching the other peaks identified with the genomic DNA samples, i.e. peaks that were entirely distinct from those identified in the other flow-sorted then amplified samples. This indicated to us that one sample was a flow-sorted and amplified chromosome 6 inherited from one parent and four samples were flow-sorted and amplified chromosome 6 inherited from the donors other parent.

SNP genotyping arrays were performed using amplification products to confirm the parental chromosome source (Fig. 3). SNPs from the diploid genomic DNA of the donor were compared to SNP calls from the whole chromosome amplification products on a SNP array. Two examples identified as single chromosome 6 samples were confirmed as being of separate parental origin, and these samples matched the donor genomic DNA with microsatellite markers. The SNP calling software designates each polymorphic base as either A or B, thereby calling a SNP as one of AA or BB (homozygotes) or AB (heterozygote). In the diploid genomic DNA sample, the 33655 SNPs probed were called as 20.0% (6755) heterozygotes AB (central cluster) with 31.2% annotated as AA (vertical cluster) and 48.6% BB (horizontal cluster) (Fig. 3). Using an arbitrary raw fluorescence units (rfu) cut-off value of 5000, sample #1 called the respective A and B SNPs uniquely to its haplotype 97.5% (2572/2627 and 2584/2665) and sample #2 assigning 97.3% (2159/2217 and 2931/3012) uniquely (Fig. 3). False heterozygotes were called at 1.48% (498/33656 SNPs) for sample #1, and 1.03% (346/33656) for sample #2, enabling SNP haplotypes to be determined for 98.5% of SNPs for sample #1 and 99.0% for sample #2. Comparatively, the Theta allele calls were made in 99.9% of the genomic DNA variants, with Sample #1 at 76.8% (25835/33655) and Sample #2 at 64.8% (21803/33655) using GenomeStudio basecalling. Of the Theta allele SNPs called as heterozygote (6650 SNPs) from the genomic DNA, 42.2% (2771 SNPs) were differential homozygote calls (i.e. AA/BB, or BB/AA) on the amplified single chromosomes, with 8.9% (599 SNPs) corresponding to bases on both amplified samples and 50.1% missing due to allele dropout. Theta allele SNPs called as homozygote on the genomic DNA, 18.9% (5100/26999 SNPs) unavailable for analysis due to non-calling of one or both amplified samples, were matched in 52.0% (14029/26999) SNPs across the two amplified samples and discordant in 29.1% of calls (Supplementary Fig. S1. It is foreseeable that optimisation of the amount of amplified single chromosome product loaded on the SNP array can markedly improve the basecalls. However, the results indicated to us that the full length of each flow-sorted-then-amplified chromosome of was of distinct parental origin and was amplified and not lost during sorting.

The two samples of single flow-sorted-then-amplified chromosome 6 that were identified as being from each parent were used to generate libraries for sequencing. To analyse the HLA region, we applied software specifically designed for HLA typing, namely Omixion HLA Typer. After sequencing (average of 44X coverage per library), sample #1 successfully called at low-resolution HLA-A^*^01, DQB1*05, HLA-DRB1*01 and for sample #2, HLA-A*01, HLA-C*07, HLA-DQB1*02, HLA-DRB1*03 corresponded with standard alleles determined by sequence based typing. To improve sensitivity on alleles unresolved in the previous analysis, depth of coverage was increased to 110X, on average, per library. As a result, sample #1 yielded 6/6 calls corresponding to sequence based typing while sample #2 yielded 5/6 of the alleles. Accuracy of sequencing reads was determined by aligning against conventional sequence based typing of the six transplant matching loci. There was a high degree of variability of depth of coverage over the key HLA regions used in transplant matching and across the haplotype (Fig. 4). For instance, the exon 2 region of MHC class II genes (*HLA-DRB1*, *HLA-DQA1*, *HLA-DQB1*, *HLA-DPA1* and *HLA-DPB1*) and exon 2 and exon 3 of MHC class I genes (*HLA-A*, *HLA-B* and *HLA-C*) are used in PCR-based matching and the gene coverage for these regions ranged from 70 to 100% (Fig. 4). The coverage of the HLA-B locus in exons 2 and 3 was particularly low for single chromosome sample #2, which was possibly a consequence of the combination of a degree of degradation of sample #2 that was also reflected in the SNP array calls, and shorter fragment lengths of amplifying *HLA-B* for the single-cell amplification kit^18^. Omixion HLA Typer failed to assign the *HLA-B* locus on single chromosome sample #2, presumably due to low coverage at exon 2. We therefore used CLC Genomics Workbench to call HLA-B alleles for both sample #1 and 2. It called sample #1 as an alternative HLA-B*27 allele HLA-B*27:05:02 (HLA-B*27:09 for Omixion HLA Typer) and sample #2 as the ambiguous group: B*08:01:01, B*08:01:04, B*08:01:29, B*08:02, B*08:108, B*08:132, B*08:29, B*08:33, B*08:93, B*41:01:01, B*41:02:01, B*41:33, B*42:01:01 or B*42:02, the : B*08 being concordant with the allele assignment via sequence based typing. Pooling sequencing runs (154X depth of coverage) did not appreciably improve allele calling, possibly indicating allele dropout at key exons during amplification. Haplotypes were assembled using allele calls from HLA Typer (Table 1). Although HLA-B*08 did not have sufficient depth of coverage to be successfully called with Omixon HLA Typer, CLC Genomics Workbench successfully resolved the allele as HLA-B*08, which was less discriminating than the assignment made by sequence based typing. Single chromosome sample #1 was identified as matching the A1-B8-DR3 haplotype^19^. As previously mentioned, the relatively low sequencing depth of coverage at the key exons of HLA-B*08 and HLA-B*27, is possibly due to dropout during amplification and requires further exploration. To validate the phasing we used HLA*IMP:02, to impute alleles directly from the HumanCoreExome array. Allele assignment of the genomic DNA was predominantly accurate, though due to the low density of the HumanCoreExome over the MHC region, *HLA-A* and *HLA-DPB1* were unable to be imputed. HLA-B*08:01, (HLA-B*27:05 called falsely as HLA-B*07:05), HLA-C*01:02,*0701, HLA-DRB1*01:01,*03:01, HLA-DQA1*01:01,*05:01, HLA-DQB1*05:01*02:01 were correctly identified. Sample 1 was phased as HLA-C*01:02, HLA-DRB1*01:01, HLA-DQA1*01:01, HLA-DQB1*05:01 with Sample 2 only resolving the HLA-DRB1*03:01 allele.

## Discussion

**Figure 1:**
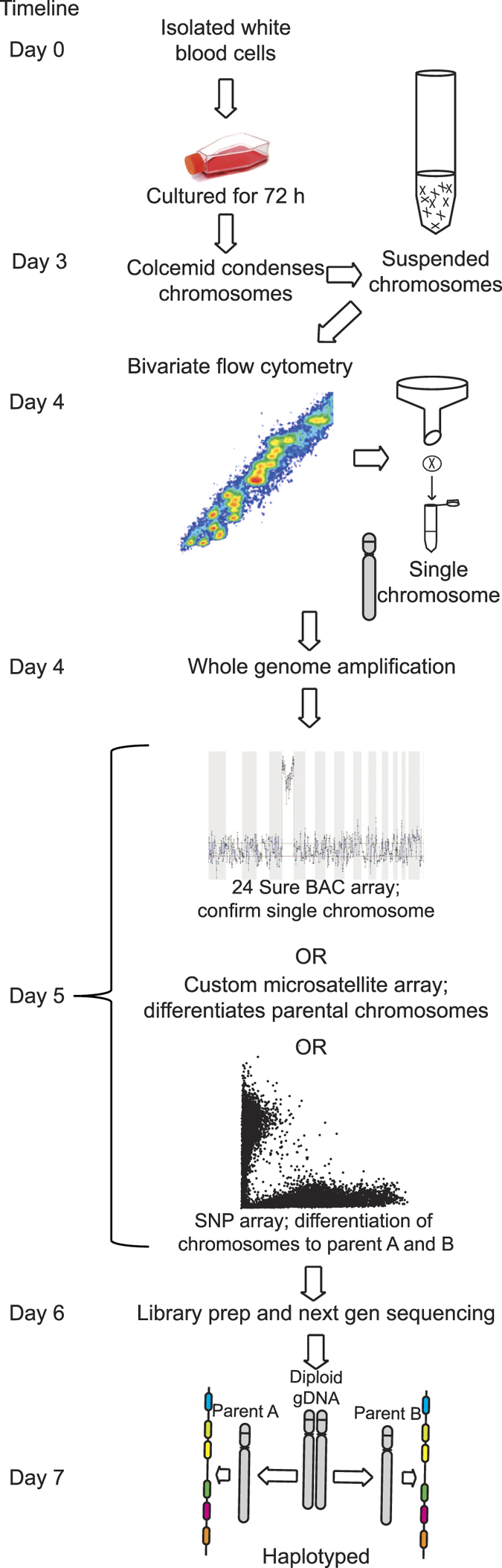
Molecular haplotyping of HLA: overview of the workflow. A patient’s white blood cells are purified and cultured. Addition of colcemid arrests cell division at metaphase, condensing chromosomes sufficiently to survive vigorous hypotonic cell lysis. Bivariate flow cytometry allows visual recognition of chromosome 6 which is then flow sorted into single PCR tubes. A whole genome amplification kit optimised for single cells yields sufficient material to confirm the presence of chromosome 6 via BAC and/or SNP array to discover the parental source, quality, and detection of contamination. Library generation and paired-end next-generation sequencing at 110X depth, yields sufficient coverage for HLA allele calling and haplotype phasing.

**Figure 2:**
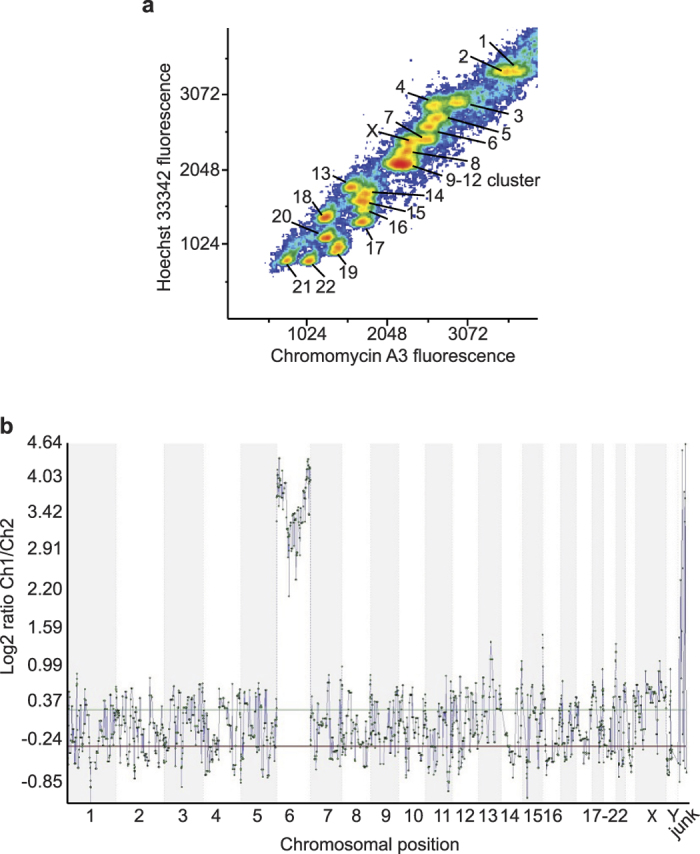
Separation of chromosomes and confirmation of isolated chromosome 6 samples. (**a**) Bivariate flow plot of single chromosome acquisition sorting. The X-axis indicates the fluorescence emitted by Chromomycin A3 specific binding to guanine-cytosine (C-G) regions of DNA, the Y-axis indicates the fluorescence emitted by Hoechst 33258 binding to adenine-thymine (A-T) regions of DNA. (**b**) 24 Sure (+) BAC microarray (BlueGnome, Cambridge) of chromosome 6 signal hybridised against male reference genomic DNA indicating the presence of chromosomal DNA from chromosome 6. Background noise for the remaining chromosomes indicates a lack of DNA from chromosomes 1–5, 7–22, X or Y.

**Figure 3:**
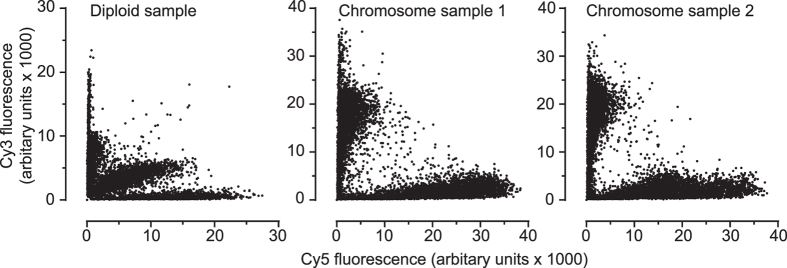
Cy3 and Cy5 fluorescence values obtained from Human Core Exome SNP array (Illumina) for 200 ng diploid gDNA (left), flow-sorted-then-amplified single chromosome 6 sample #1 (centre), and flow-sorted-then-amplified single chromosome 6 sample #2 (right).

**Figure 4:**
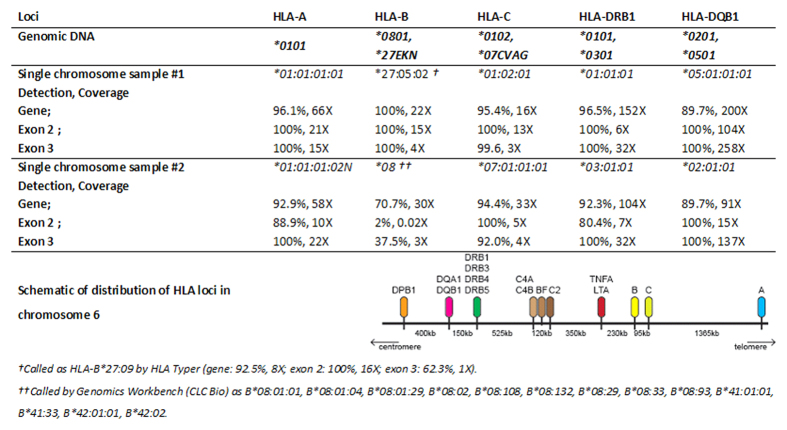
Allele typing of transplant-associated loci using sequence specific typing (genomic DNA) compared to single chromosome amplification and sequencing of the isolated parental chromosome 6 samples. Alleles were confirmed by sequencing. The loci used are within exon 2 and 3 of HLA Class 1 genes (*HLA-A*, *HLA-B* and *HLA-C*) and HLA Class II genes (*HLA-DRB1*, *HLA-DQA1*, *HLA-DQB1*, *HLA-DPA1* and *HLA-DPB1*). The percentage values are the percentage of the gene/feature covered by the aligned sequence data. Therefore, it is possible for exon 2 and 3 to be 100% covered, but the gene to only be 89.7% covered, due to poor coverage in another part of the gene.

To fully validate the haplotypes, a family study would provide additional depth to the study^8^. The data presented here indicating highly disparate SNP calls and HLA allele imputation gives confidence that the two chromosomes selected for high throughput sequencing were of separate parental origin, but also emphasises the limitations of SNP arrays for precise phasing. Chromosome separation offers genuine phasing and there is no possibility of recombination between the two chromosomes during the amplification steps. A further advantage of chromosome separation is that phasing is chromosome wide and therefore haplotypes do not need to be imputed or rely on typing parental samples. One of the haplotypes identified in this study was the most common Caucasian haplotype A1-B8-DR3-DQ2^19^,^20^, which simplified analysis. With the allele calling of the sequence-based typing contiguous to the high throughput sequencing allele calls, the combined results indicate proof of successful haplotype phasing of HLA. A turnaround time of approximately one week from blood extraction to phased sequenced data is feasible using this approach (Fig. 1) and highly desirous for urgent cases in transplantation laboratories.

The method described addresses a crucial problem in clinical HSCT when using unrelated donors, which is the ability to discriminate between allele-matched or haplotype-matched donor-recipient pairs. Despite evidence that haplotype matching gives superior clinical outcomes there is currently no method that can routinely provide haplotype matching^21^. An added benefit of the approach described in this study is the ability to unambiguously define extended haplotypes which include polymorphic non-HLA genes and future directions of the research could include measurement of linkage disequilibrium between the observed SNP array calls and the HLA alleles assigned. Conversely, the high degree of redundancy in the number of unused reads in order to elucidate loci such as *HLA-B* indicates that approaches aimed to either specifically amplify the MHC or limit the amplification of the non-MHC sequences would increase the economy of the sequencing depth of coverage for the desired regions. Alternatively, amplification of single chromosomes using long-range amplification followed by locus-specific PCR and sequencing would allow alleles to be individually called and haplotypes generated in the context of transplant HLA-matching^22^. The approach applied here has been previously proposed as an effective means to produce a fully phased genome^1^. Phasing by dilution to ultra-low copy numbers has been shown as an effective method for genome phasing^23^. However, due to the depth of sequencing required to resolve alleles indicated in this study, phasing by dilution may not be well-suited to directly haplotyping HLA.

In conclusion, this study has shown that complete HLA haplotype phasing can be achieved by amplifying single chromosomes and performing deep high-throughput sequencing. This study attempted to address the difficulty of assigning HLA haplotypes in the absence of family studies by applying an established but under-utilised technique, which if developed further, would be of immense benefit to the broader landscape of genetics. Implications that flow from this study extend far beyond the field of clinical transplantation and include the ability to phase multiple SNPs across the genome. This would provide insight into SNPs associated in a range of diseases where SNP phase has not been determined. Reporting of haplotype information will provide clinicians, patients and medical researchers with a better understanding of cis and trans-interactions in functional polymorphisms and their impact on disease susceptibility and progression, leading in turn to more informed treatment options.

## Methods

### Human blood samples

All experimental protocols involving human blood samples were approved by the ethics committee of the Australian Bone Marrow Donor Registry (ABMDR: Project 2011/03). Methods for handling of blood samples were carried out in accordance with the approved guidelines. Informed consent was obtained from all subjects by the ABMDR.

### Chromosome suspension

The blood sample- (50 mL) was collected in lithium heparin tubes from an individual registered with the Australian Bone Marrow Donor Registry (ABMDR). To generate a chromosome suspension directly from blood, white cells were separated by a density gradient centrifugation method using Histopaque 1077 (Sigma USA) and centrifugation at 1000 × g for 10 min. White blood cells were then aspirated with a Pasteur pipette and washed twice by resuspending in 50 mL phosphate buffered saline (PBS) and centrifuged at 250 × g for 5 min and once in 50 mL of RPMI 1640 (Life Technologies, USA). The pellet was then suspended in 40 mL RPMI 1640 with 1 mL phytohemagglutinin (Gibco, USA) and 100 μL of fetal calf serum in a T75 flask and cultured at 37 °C, 5% CO_2_ for 72 hours. 400 μL of 10 g/mL colcemid (Life Technologies, USA) was added and after 6 hours, the cells were harvested by washing twice with PBS and centrifuging at 250 × g for 5 mins. The pellet was gently suspended in 15 mL of 0.075 M KCl solution at 37 °C for 20 mins followed by centrifugation at 250 × g for 5 mins. The pellet was gently suspended in freshly prepared 3 mL of ice-cold polyamine buffer (0.5 mM spermidine (Sigma), 0.2 mM spermine (Sigma), 5 mM EGTA, 20 mM EDTA, 150 mM Tris, 0.25% Triton-X 100, 30 mM DTT and 10 mM MgSO_4_)^24^. After 5 mins the cell suspension was vigorously vortexed for 15 seconds, releasing chromosomes into suspension. The suspension was then stored at 4 °C for subsequent sorting by bivariate flow cytometry.

### Chromosome sorting

In order to identify the chromosome clusters, 16 hours before flow cytometry, 10 μL of 1 mg/mL DNA stain Hoechst 33258 and 32 μL of 2.5 μg/mL of Chromomycin A3 (that preferentially bind adenine-thymine and guanine-cytosine rich DNA sequences respectively), were added to 2 ml of chromosome suspension. One hour prior to sorting 10 μL sodium citrate (1 M) and 50 μL of sodium sulphite (500 mM) was added to facilitate chromosome condensing and stability, prior to filtering through a 20 μm filter (Partec) to eliminate cellular debris. The flow karyotype was generated on a MoFlo cell sorter (Beckman Coulter) using a 70 μm nozzle and a sheath pressure of 56 psi. Chromomycin A3 was excited with an Argon Ion laser tuned to 457.9 nm with power set to 300 mW. Hoechst 33258 was excited with a solid state laser emitting at 355 nm with power set at 300 mW. Chromomycin A3 fluorescence was collected using a 490LP filter and Hoechst 33258 fluorescence with a 400SP and 457.9 nm notch laser blocking filter. A sorting gate was placed over the chromosome 6 cluster and a single chromosome 6 was sorted into each PCR tube.

### Single chromosome amplification

DNA amplification of the single chromosomes was performed using the single cell whole genome amplification PicoPLEX Kit (Rubicon Genomics, USA) as per manufacturer’s instructions. Half of the amplification product was then purified using a GeneJet PCR purification kit (Thermo Scientific, USA) for library preparation, and the non-purified product was retained for SNP array testing.

### BAC Array

24 Sure (+) BAC microarray (BlueGnome, Illumina, USA) was used for single chromosome 6 screening, described by Tobler *et al*.^25^. Briefly, DNA samples were labelled with random primers, hybridised to a BAC microarray with Cy3, scanned on InnoScan 710 (Innopsys) and reference compared against a euploid male labelled with Cy5. Raw fluorescence was measured by Mapix (Innopsys) and signal strength normalised into the presence of chromosomal DNA by Bluefuse Multi (Bluegnome, Illumina, USA).

### SNP Array

200 ng of the amplified product was used on the HumanCoreExome BeadChip array. An arbitrary cut-off value for SNP calls was set at ≥5000 relative fluorescent units (rfu), i.e. ‘AA’ if fluorescence >5000 rfu for ‘A’ base and <5000 rfu for ‘B’ base, ‘AB’ if fluorescence >5000 rfu for ‘A’ and ‘B’ base, ‘BB’ if fluorescence <5000 rfu for ‘A’ base and >5000 rfu at ‘B’ base.

### Sequencing

Amplified products selected for library preparation were quantified via Tapestation (Aglient, USA) sequencing using Quant-IT PicoGreen (Invitrogen, USA) against a lambda DNA standard. Libraries of 1 ng of dsDNA product were used with the Nextera XT DNA Sample Preparation kit (Illumina, USA), size selected at 444 bp (from 214 – 775 bp) and 392 bp (from 167–737 bp) for each sample and sequenced using 100 bp paired-end on a HiSeq2500 (Illumina, USA). Sequencing on a single lane yielded 96,839,980 and 103,733,615 paired reads for the two libraries.

### Software

HLA typer (Omixon) was used to assemble paired-end reads against reference HLA-alleles^26^. Alleles were called for each loci as the allele having the highest mean depth of coverage across each gene region. The maximum fragment size was fixed at 600 bp, multi-mapping, *i. e.* reads that mapped to multiple genes were discarded, as were rare alleles. For the *HLA-B* gene, sequencing reads were also aligned with CLC bio genomics workbench (CLC Genomics Workbench 7.3 (http://www.clcbio.com)). In house software (HLA Allelecaller) used these alignments combined with the reference data in the international ImMunoGeneTics information system (IMGT) database to assign all alleles concordant with the data from exons one to four inclusive^27^. HLA*IMP:02 was used to perform allele imputation from HumanCoreExome BeadChip array data^28^. The genotyping results from the HumanCoreExome-12v1-0_B were exported as PLINK input report files from Illumina GenomeStudio (v2010.4.0.301128). The ped and map files were read into PLINK (v1.07)^29^. The –make-bed command was used to create a binary file. This file was read into PSEQ^30^ and subsequently exported as a VCF. The VCF file was edited to VCF format v4.0 and read into the HLA*IMP front end. The file underwent conversion to oxford HLA standard format, and missing data was removed (with a threshold of 0.2). The resulting file was loaded into the web interface of HLA*IMP and all loci except HLA A and HLA DPB1 were imputed.

## Additional Information

**How to cite this article**: Murphy, N. M. *et al*. Haplotyping the human leukocyte antigen system from single chromosomes. *Sci. Rep.* **6**, 30381; doi: 10.1038/srep30381 (2016).

## Supplementary Material

Supplementary Information

## Figures and Tables

**Table 1:** Allele typing of the individual generated by HLA Typer (Omixon).

Locus	Sample #1	Sample #2
*HLA-F*	*01:01:03:01 (64X)	*01:01:03:01 (64X)
*HLA-V*	*01:01:01:01 (20X)	*01:01:01:01 (32X)
*HLA-G*	*01:01:02:01 (105X)	*01:01:02:01 (80X)
*HLA-H*	*02:01:01:01 (88X)	*02:01:01:01 (69X)
*HLA-K*	*01:01:01:01 (114X)	*01:01:01:01 (63X)
*HLA-A*	*01:01:01:01 (64X)	*01:01:01:02N (58X)
*HLA-J*	*01:01:01:01 (86X)	*01:01:01:02 (71X)
*HLA-L*	*01:01:02 (109X)	*01:01:02 (47X)
*HLA-E*	*01:01:01:01 (219X)	*01:01:01:01 (218X)
*HLA-C*	*01:02:01 (16X)	*07:01:01:01 (32X)
*HLA-B*	*27:05:02 (22X)	*08 (30X)
*MICA*	*001 (112X)	*008:01:01 (94X)
*MICB*	*005:02:02 (106X)	*005:02:02 (115X)
*HLA-DRA*	*01:01:02 (60X)	*01:02:02 (83X)
*HLA-DRB9*	*09:01 (33X)	*01:01 (30X)
*HLA-DRB2*	—	*01:01 (46X)
*HLA-DRB3*	—	*01:01:02:01 (109X)
*HLA-DRB6*	*06:01 (2X)	—
*HLA-DRB1*	*01:01:01 (152X)	*03:01:01 (104X)
*HLA-DQA1*	*01:01:01 (52X)	*05:01:01:02 (108X)
*HLA-DQB1*	*05:01:01:02 (200X)	*02:01:01 (91X)
*HLA-DOB*	*01:01:03:01 (169X)	*01:01:01:02 (191X)
*HLA-TAP2*	*01:01:02 (136X)	*02:01:02:01 (126X)
*HLA-TAP1*	*03:01 (202X)	*01:02N (112X)
*HLA-DMB*	*01:01:01:01 (87X)	*01:01:01:02 (81X)
*HLA-DMA*	*01:01:01:01 (33X)	*01:01:01:02 (42X)
*HLA-DOA*	*01:01:02:03 (85X)	*01:01:05 (81X)
*HLA-DPA1*	*01:03:01:05 (80X)	*02:01:02 (78X)
*HLA-DPB1*	*04:02:01:02 (306X)	*01:01:01:01 (35X)

Numbers in brackets indicate the depth at which the mean coverage was assigned per locus.
