# Supplementary material for: Haplotyping the human leukocyte antigen system from single chromosomes
Source: Sci Rep. 2016 Jul 27;6:30381. doi: 10.1038/srep30381 (PMC4961964; doi:10.1038/srep30381)
Supplement: Supplementary Information [file srep30381-s1.pdf]

Supplementary Information

Haplotyping the human leukocyte antigen system from single chromosomes

Nicholas M. Murphy, Matthew Burton, David R. Powell, Fernando J. Rossello, Don Cooper, Abha Chopra, Ming Hsieh, David C. Sayer, Lavinia Gordon, Mark D Pertile, Brian D. Tait, Helen R. Irving, Colin W. Pouton

a

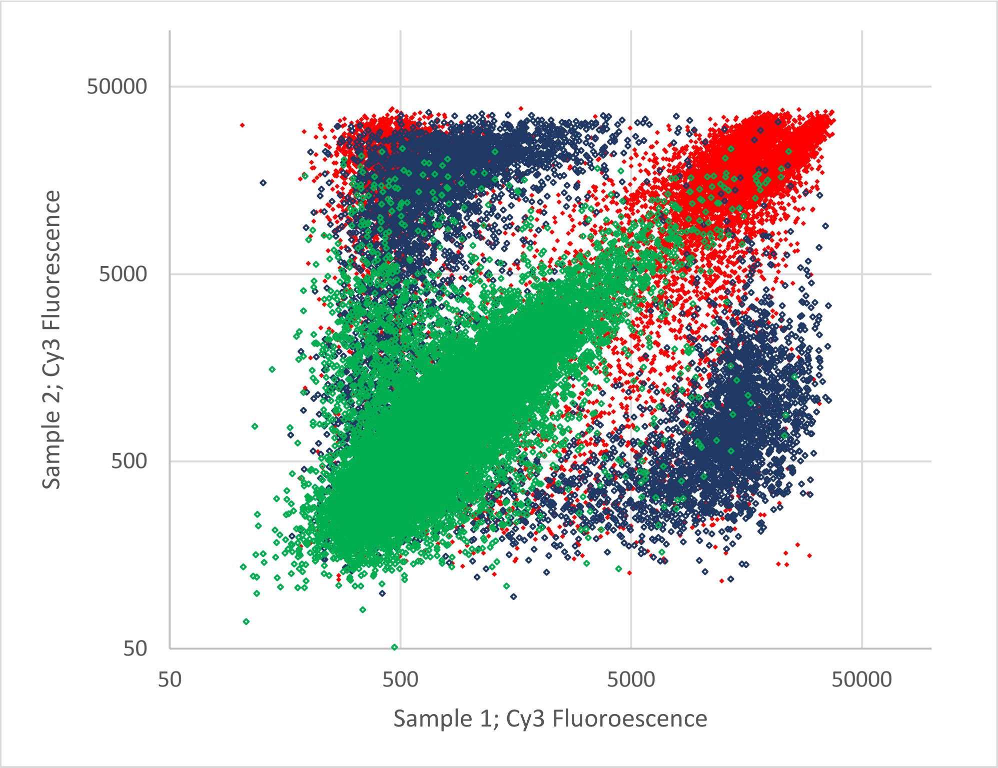

b

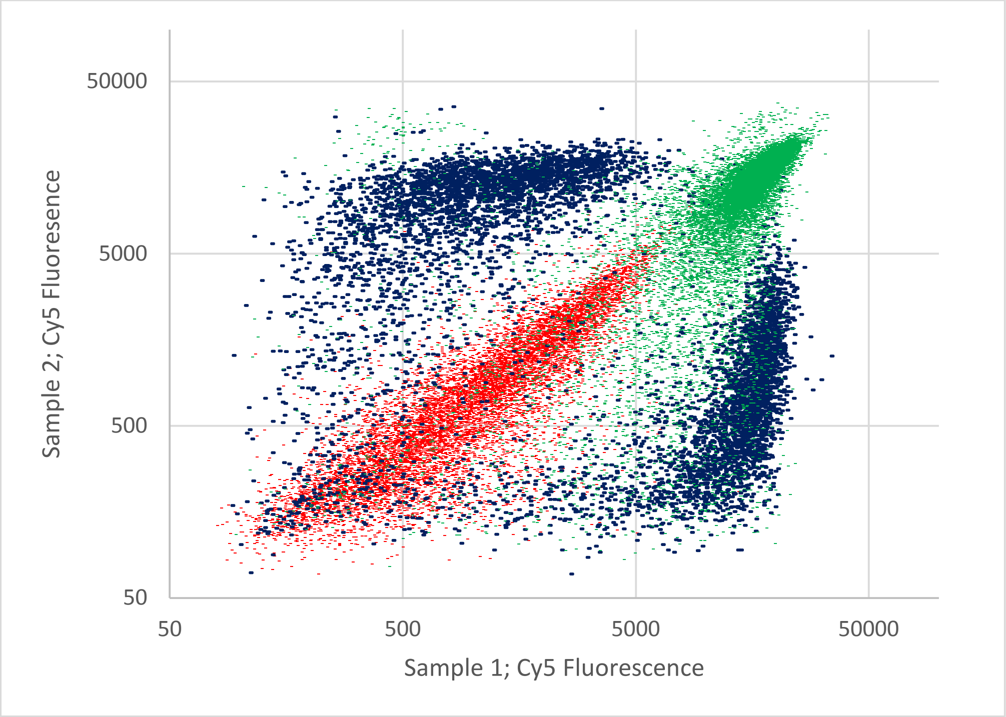

Supplementary Figure S1. Raw fluorescence values of two amplified single chromosomes (Sample 1, a; Sample 2,) of the HumanCoreExome array plotted on opposing axis which have been split into the allele calls made by GenomeStudio on the genomic DNA. Green signals indicate heterozygote ‘AB’ calls, red signals indicate homozygote ‘AA’ calls, blue signals indicate homozygote ‘BB’ calls for Cy3 fluorescence (a) and Cy5 fluorescence (b).
